# A case of Mac Tel 2 with an unusual sub macular vitelliform lesion

**DOI:** 10.3205/oc000031

**Published:** 2015-10-19

**Authors:** T. Lekha, Nikit Sarwate, Renuka Sarwate

**Affiliations:** 1 Department of Ophthalmology, PSG Institute of Medical Sciences and Research, Coimbatore, Tamilnadu, India; 2 Retina Services, The Eye Foundation, Coimbatore, Tamilnadu, India

**Keywords:** Mac Tel 2, vitelliform lesion, optical coherence tomography, mfERG

## Abstract

**Method:** Observational case report describing the clinical, FFA, OCT and mfERG findings in an elderly female patient with atypical features of macular telangiectasia (Mac Tel 2)

**Results:** A 71-year-old lady was detected to have characteristic features of Mac Tel 2 in the left eye (LE) and a yellowish sub macular vitelliform like lesion in the right eye (RE). FFA showed ill defined hyper fluorescence in the RE and telangiectasia and parafoveal leakage typical of Mac Tel 2 in the LE. On OCT RE had hyper reflective clump of echoes subfoveally with an intact RPE and LE had foveal thinning with hypo reflective intraretinal cavities. mfERG responses were normal in the RE and reduced in the LE. During the course of 3 years LE showed natural progression while RE remained unchanged.

**Conclusion:** Structural and functional evaluation of an unusual sub macular vitelliform lesion seen in association with Mac Tel 2 and its course over a period of 3 years is described. The differentiating features of this lesion from adult onset foveomacular vitelliform dystrophy (AFMD) are discussed.

## Introduction

Macular telangiectasia (Mac Tel 2) or Idiopathic Para Foveal Telangiectasia (PFT) is now recognized as a primary neuroretinal degenerative condition with secondary vascular involvement [1], [2]. It is typically a bilateral, symmetrical disease characterized by decrease in retinal transparency, telangiectasias, refractile deposits in the superficial retina, hyperpigmented RPE plaques and subretinal neovascularization [3]. Rarely small, round yellow lesions may be seen in the fovea mimicking the vitelliform lesions in Adult onset foveo macular dystrophy (AFMD) [4], [5], [6]. Spectral domain Optical Coherence Tomography (OCT) is valuable in distinguishing the two conditions [7]. We describe the case of an elderly woman with typical PFT in the left eye (LE) and a vitelliform like lesion in the right eye (RE). She was evaluated by Fundus Fluorescein Angiography (FFA), OCT, multifocal electroretinography (mfERG) and followed up for 3 years. To the best of our knowledge this is the first report describing the structural and functional evaluation of such a rare lesion. 

## Case description

**Figure 1:**
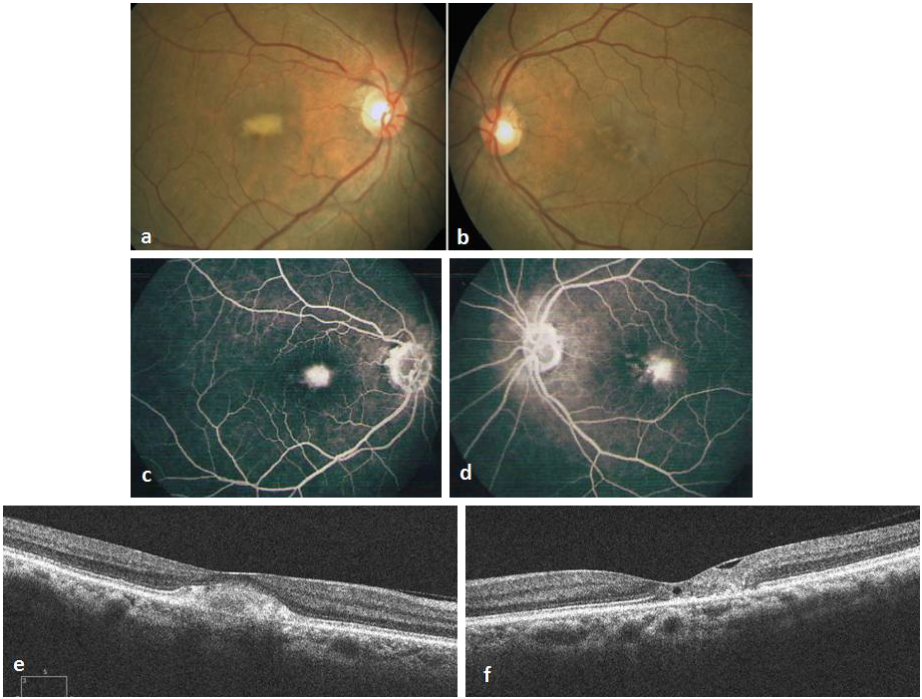
Fundus photograph at presentation showing sub macular yellowish lesion, one-third disc diameter in size, not associated with any vascular changes, pigmentation or CNV in the RE (a), RPE hypo pigmentation over fovea with minimal graying and telangiectasia in the LE (b). Fluorescein angiography showing ill defined hyper fluorescence sub foveally in the RE (c) and temporal parafoveal leakage in the LE (d). Spectral domain Optical Coherence Tomography (OCT) of the RE showing hyper reflective clump of echoes sub foveally, normal morphology of all the retinal layers around the lesion and no features of CNV (e). In the LE there is foveal thinning, small sub foveal lamellar hole and internal limiting membrane (ILM) drape with disruption of outer retinal layers temporally (f). Disruption of the ellipsoid line and intact RPE are noted in BE.

**Figure 2:**
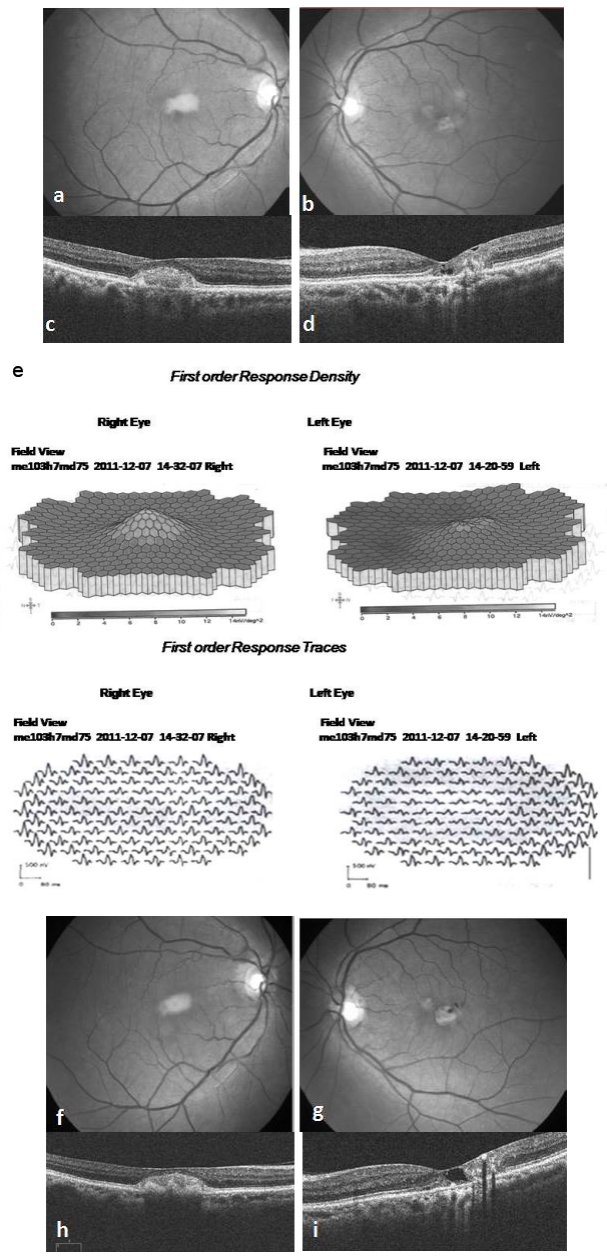
At 15 months follow up the sub retinal lesion in the RE remaining unchanged on fundus (a) and OCT (c) whereas LE fundus showing increased parafoveal RPE depigmentation and a small pigmented plaque along the right angled venule temporally (b). OCT of the LE showing multiple lamellar holes sub foveally increased disruption of the outer retinal layers with hyper reflective echoes temporally suggestive of progression (d). mfERG recording (e) showing normal response in the RE and diminished amplitudes in the inner rings in the LE. At her final visit at 3 years, no significant changes are seen ophthalmoscopically or on OCT in the RE (f, h), but LE is showing further progression with increased RPE atrophy and pigmented RPE plaques (g). OCT showing enlarged lamellar hole and RPE hyperplasia causing shadowing temporal to fovea (i).

A 71-year-old healthy woman presented to our hospital in September 2010 with a referral diagnosis of choroidal neovascularization (CNV) in the RE for which she was advised intravitreal Bevacizumab injection. On examination she had a best corrected Snellen’s visual acuity (BCVA) of 6/12 in the RE, 6/9 in the LE, normal color vision in both eyes (BE) and immature cataract in BE. Fundus of RE had a yellowish sub macular lesion of one-third disc diameter in size. LE had retinal pigment epithelial (RPE) depigmentation and minimal telangiectasia parafoveally. (Figure 1 a, b (Fig. 1)). FFA showed ill defined hyperflourescence subfoveally in the RE and temporal parafoveal leakage in the LE (Figure 1 c, d (Fig. 1)). OCT of RE revealed subfoveal hyper reflective clump of echoes, discontinuity of the ellipsoid line (IS/OS layer) with intact RPE. The central foveal thickness (CFT) was 270 microns. OCT of LE showed foveal thinning with CFT of 180 microns, multiple lamellar holes, break in the IS/OS layer and intact RPE (Figure 1 e, f (Fig. 1)). A diagnosis of PFT in the LE and a vitelliform like lesion in the RE was made. As there was no sign of CNV only observation was advised. By December 2011, her BCVA had reduced to 6/24 in the RE and 6/12 in the LE. Fundus and OCT remained unchanged in the RE, but LE showed increased RPE depigmentation and pigmented plaque along the dilated venule (Figure 2 a–d (Fig. 2)). At this stage the rare possibility of co-existent AFMD in the RE was suspected, but was ruled out after normal electroretinography (ERG) and electrooculography (EOG) recordings in BE. Arden’s ratio was 2.7 in the RE and 4.3 in the LE. mfERG amplitudes were normal in the RE and diminished in the inner rings in the LE (Figure 2 e (Fig. 2)). At her next visit in July 2013 BCVA had deteriorated to 6/36 in the RE and 6/18 in the LE with worsening of cataract in BE. Following cataract surgery in the LE her BCVA improved to 6/9. Fundus and OCT evaluation showed RE to be status quo whereas there was progressive RPE atrophy, pigmented plaques and cavitation in the LE (Figure 2 f–i (Fig. 2)). She was advised cataract surgery under guarded visual prognosis in the RE, but was lost to follow up.

## Discussion

Mac Tel 2 is often under diagnosed in the early stages due to subtle fundus changes or misdiagnosed in the late stages as age related macular degeneration resulting in inappropriate treatment [1]. Advent of spectral domain OCT and other techniques of multimodality imaging have facilitated earlier and accurate diagnosis. However diagnosis can still be challenging in eyes with unusual features such as vitelliform lesions as observed in our patient.

Yellowish foveal lesion first documented by Gass in eyes with PFT can be mistaken clinically for AFMD [7]. Similar lesions reported by other authors as pseudovitelliform lesions could have been attributed to PFT if structural evaluation by OCT were possible [4], [5], [6]. The OCT features of such lesions include hyper reflective mound of material between the ellipsoid region and RPE with irregular reflectivity and focal loss of the overlying photoreceptor layer and outer limiting membrane [7]. AFMD is also characterized by similar hyper reflective sub retinal lesions resulting from shed photoreceptor outer segments [8]. However in AFMD there is obvious reaction to the material with RPE hypertrophy, hyper pigmentation or anterior migration whereas in PFT, RPE remains as a monolayer with minimal reaction, probably due to defective muller cell function. In AFMD phagocytosis of these outer segments may occur eventually, loading the RPE cells with increased amount of lipofuscin, a feature which has been proven pathologically [9].

Other investigations are also useful in differentiating the subretinal lesion in Mac Tel 2 from AFMD. FFA shows early hypo fluorescence and late staining in AFMD, but no distinguishing features are seen in Mac Tel 2 [7]. Full field ERG may be normal in both but mfERG is reduced in the macula and in the periphery in AFMD indicating widespread RPE abnormality [10]. mfERG studies in PFT indicate moderate reduction in the amplitude and increased implicit time suggestive of early damage to bipolar cells, which is hypothesized to be due to depletion of xanthophyll pigment [11]. However mfERG responses specific to subretinal debris has not been described so far.

Our patient with typical PFT in the LE showed natural progression over 3 years, whereas the subretinal lesion in the RE remained unchanged both clinically and on OCT. FFA and OCT features of the sub macular lesion in our patient is similar to that described in the literature. Reduced mfERG amplitudes in the LE were expected but normal amplitudes in the RE indicate relatively intact macular function despite the sub retinal debris. These observations along with the lack of progressive structural changes with time probably indicates an attenuated RPE response and defective muller cell function to be responsible for the accumulation of photoreceptor outer segments in the subretinal space, which in turn may have a role in preventing progression of the disease. Based on the analysis of a single eye it is difficult to comment whether such subretinal debris is masking the typical findings or it actually prevening the progression of PFT. Another limitation is that only the OCT changes and not the mfERG features could be evaluated over time. Also if newer imaging tools like adaptive optics, confocal blue reflectance imaging and fundus auto fluorescence were available, it would have provided more insight into the nature of such rare lesions. 

## Notes

### Competing interests

The authors declare that they have no competing interests.

### Acknowledgements

Dr. D. Ramamurthy, Chairman, The Eye Foundation, Coimbatore, Tamilnadu, for institutional and equipment support
